# Microfluidic Electrochemical Biosensor for the Detection of Platelet Factor 4 Antibody‐Mediated Thrombotic Disorders

**DOI:** 10.1002/advs.202513607

**Published:** 2025-12-19

**Authors:** Diana F. Cedillo‐Alcantar, Adam Kanack, Seonhwa Lee, Alan M. Gonzalez‐Suarez, Kihak Gwon, Emily Mauch, Thi Thanh‐Qui Nguyen, Anand Padmanabhan, Alexander Revzin

**Affiliations:** ^1^ Department of Physiology and Biomedical Engineering Mayo Clinic Rochester MN 55902 USA; ^2^ Department of Laboratory Medicine and Pathology Mayo Clinic Rochester MN 55902 USA; ^3^ Department of Biofibers and Biomaterials Science Kyungpook National University Daegu 41566 Republic of Korea

**Keywords:** biosensors, heparin‐induced thrombocytopenia, microfluidics, vaccine‐induced immune thrombotic thrombocytopenia

## Abstract

Vaccine‐induced immune thrombotic thrombocytopenia (VITT) is a new disorder that emerged in the wake of coronavirus disease 2019 (COVID‐19) vaccination. It is a rare but life‐threatening condition that requires an aggressive course of treatment to improve patient outcomes. Currently, the diagnosis of VITT relies on a comprehensive panel of criteria, including a history of recent vaccination, platelet count, positive enzyme linked immunosorbent assays (ELISA) result for a closely related thrombotic disorder, heparin‐induced thrombocytopenia (HIT), and platelet factor 4 (PF4)‐dependent functional assays. The study describes a technically simple antigenic assay designed to detect autoimmune antibodies associated with VITT. It is first shown that cross‐linked platelet factor 4 (c‐PF4) represents an antigenic target specific for VITT/VITT‐like Abs. This antigenic target is then incorporated into a microfluidic electrochemical biosensor and specific and sensitive detection of VITT/VITT‐like antibodies is demonstrated in a fully automated manner while using microliter volumes of patient sera. Fifty‐five serum samples are tested using the microfluidic electrochemical biosensor and demonstrated high accuracy for the detection of VITT sera and VITT‐like monoclonal gammopathy of thrombotic ignificance (MGTS) antibodies compared to healthy controls and HIT patients. It is envisioned that the microfluidic electrochemical biosensor will be used in combination with other clinical criteria to enable timely, sensitive, and specific diagnosis of VITT.

## Graphical abstract

A microfluidic electrochemical biosensor enables sensitive and specific detection of antibodies associated with vaccine‐induced immune thrombotic thrombocytopenia (VITT) and heparin‐induced thrombocytopenia (HIT). The platform uses two antigen targets— platelet factor 4 (PF4) and cross‐linked‐PF4 (c‐PF4)—and microliter volumes of patient sera to identify disease‐specific antibodies with high accuracy. This microfluidic biosensor approach offers a simple solution for the rapid diagnosis of immune‐mediated thrombotic disorders.

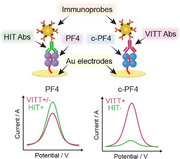

## 1 Introduction

Vaccine‐induced immune thrombotic thrombocytopenia (VITT) is a rare but severe thrombotic disorder most often caused by an immunologic reaction to adenoviral vector‐based COVID‐19 vaccines,^[^ ^1^, ^2^, ^3^ ^]^ and rarely after other types of COVID and non‐COVID‐19 vaccinations (e.g., an Human Papillomavirus vaccine).^[^ ^4^, ^5^ ^]^ VITT is characterized by the production of pathogenic antibodies (Abs) with specificity for platelet factor 4 (PF4)^[^ ^6^ ^]^ that trigger platelet activation and frequently result in thrombocytopenia and thrombosis.^[^ ^2^, ^7^ ^]^ The Ab‐mediated platelet activation associated with VITT also resembles that of other thrombotic disorders mediated by anti‐PF4 Abs, such as heparin‐induced thrombocytopenia (HIT), a disorder that frequently occurs among patients anticoagulated with heparin^[^ ^8^ ^]^ and the more recently described monoclonal gammopathy of thrombotic significance (MGTS), where patients produce monoclonal anti‐PF4 antibodies.^[^ ^9^, ^10^, ^11^, ^12^ ^]^ VITT‐like antibodies have also been noted after adenoviral infection.^[^ ^13^ ^]^

Without early detection and prompt management, HIT and VITT are associated with mortality rates of ≈10% and ≈20%, respectively. Making a timely diagnostic distinction between HIT and VITT is important: HIT is managed by cessation of heparin anticoagulation and initiation of non‐heparin alternative anticoagulation,^[^ ^14^ ^]^ while VITT patients require more aggressive treatment using intravenous immunoglobulin in addition to anticoagulation therapy.^[^ ^15^ ^]^ Additionally, there are no U.S. Food and Drug Administration (FDA)‐approved diagnostic assays to differentiate HIT‐like and VITT‐like Abs. VITT‐like anti‐PF4 antobodies (Abs) are generally less well characterized relative to HIT‐like antibodies due to their more recent discovery, but they can typically be detected using at least two types of laboratory assays: antigen‐based immunoassays and platelet‐based functional testing. Majority of established assays fail to differentiate HIT and VITT‐like Abs,^[^ ^16^, ^17^, ^18^ ^]^ however, a limited number of recent studies report success in this regard.^[^ ^19^, ^20^ ^]^ Assays reported to date have drawbacks. Platelet‐based functional assays are technically challenging and require specialized reference laboratories and freshly drawn donor platelets.^[^ ^21^ ^]^ PF4‐based functional assays have been shown to reliably detect VITT‐like antibodies but are still not specific for these antibodies.^[^ ^22^ ^]^ Additionally, enzyme linked immunosorbent assays (ELISAs) employing PF4/polyanion complexes as an antigenic target^[^ ^21^ ^]^ have a low positive predictive value for differentiating non‐pathogenic anti‐PF4 Abs from pathogenic HIT Abs that stimulate platelet activation. This necessitates confirmatory testing in functional platelet assays after a positive ELISA result.^[^ ^14^ ^]^ Most existing ELISAs for HIT and VITT detection require manual handling and are performed in a laboratory setting.^[^ ^23^ ^]^ Automated assays capable of distinguishing VITT‐ from HIT‐like antibodies will benefit timely diagnosis and treatment of anti‐PF4 antibody‐mediated disorders.

The objective of our study was to address this need by developing an immunoassay for specifically yet sensitively detecting VITT Abs. We wanted this immunoassay to be rapid and automated to make it suitable for a frontline or point‐of‐care setting to improve diagnostic efficiency. Our team has recently described an electrochemical immunoassay using gold nanoparticles (AuNP) functionalized with Abs and doped with metal ions to detect surface markers on extracellular vesicles.^[^ ^24^, ^25^ ^]^ These nanoparticles (also called immunoprobes) offered two key advantages enabling sensitive detection of a target analyte: 1) each AuNP is decorated with multiple Abs, creating multivalent interactions that enhance target affinity, and 2) each AuNP is estimated to contain 10^4^ metal ions, amplifying the electrochemical redox signal.^[^ ^25^ ^]^

We reasoned that automating the workflow of the electrochemical immunoassay while using minute quantities of sample will make the assay particularly well‐suited for the point‐of‐care setting. Fluid handling in microfluidic devices can be achieved through various strategies, including capillary valves,^[^ ^26^ ^]^, centrifugal microfluidics,^[^ ^27^ ^]^ digital microfluidics based on electrowetting,^[^ ^28^ ^]^ and pneumatic microvalves.^[^ ^29^ ^]^ All of these strategies have strengths and weaknesses. Capillary valves rely on surface tension and channel geometry to control fluid flow; however, they may be limited by suboptimal temporal control, and dependence on the fluid's physicochemical properties, which complicates their use in complex automated protocols.^[^ ^26^ ^]^ Centrifugal microfluidics, which uses rotating discs to drive flow via centrifugal force, allows multiple steps to be executed without external pumps, but relies on mechanical motion, offers limited fine flow control, and is difficult to integrate with biosensors.^[^ ^30^ ^]^ Digital microfluidics based on electrowetting provides precise droplet manipulation through electric fields and is well‐suited for miniaturized low‐volume applications; however, it requires specific dielectric coatings and high voltages, can be sensitive to evaporation, and may pose compatibility issues with certain biological fluids or reagents.^[^ ^31^, ^32^ ^]^ Automation using pneumatic microvalves offers highly precise and programmable flow control, enabling reliable sequential or parallel operation of multiple steps. These microvalves, typically integrated into multilayer polydimethylsiloxane (PDMS) devices, allow dynamic reconfiguration of protocols via software, have response times on the order of milliseconds, and may be used to handle microliter volumes.^[^ ^33^, ^34^, ^35^, ^36^ ^]^ Our team has employed PDMS devices integrating pneumatic microvalves to isolate plasma and detect biomarkers based on 5 µL of whole blood^[^ ^37^ ^]^ and to perform high‐throughput serology assays.^[^ ^38^ ^]^ Some of the limitations of the devices with pneumatic microvalves include solvent uptake into PDMS, limited scalability due to the thermosetting properties of PDMS and the complexity of multi‐layer soft‐lithography fabrication. However, several recent reports describe the integration of pneumatic microvalves into thermoplastic devices that are scalably fabricated with milling or molding.^[^ ^36^, ^39^ ^]^ Therefore, microfluidic devices with pneumatic microvalves have a clear path to scalable fabrication and commercialization.

**Figure 1:**
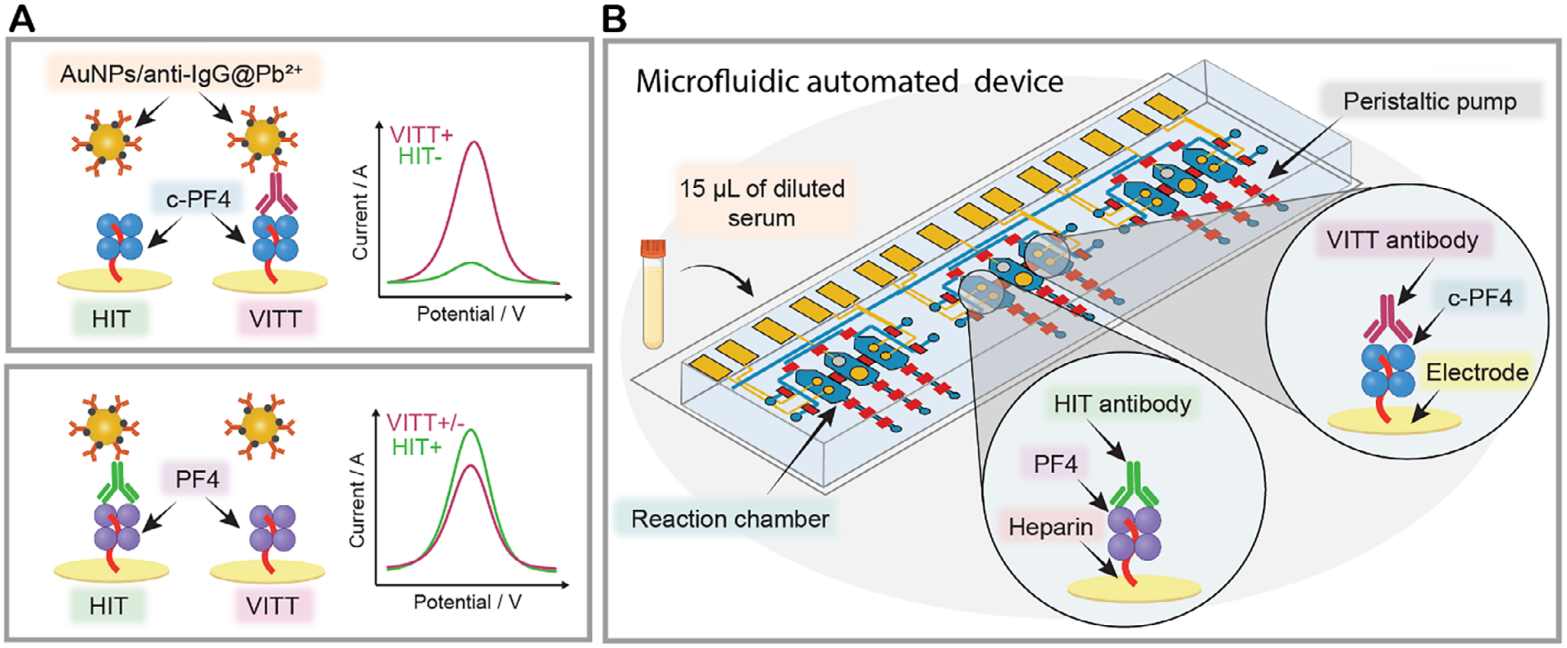
Overview of the microfluidic electrochemical biosensor. A) A sensing strategy developed in this study involves immobilizing thiolated heparin /PF4 complexes on Au electrodes. Some of the electrodes are functionalized with cross‐linked PF4 (c‐PF4) molecules that are specific to VITT Abs, while other electrodes carry uncross‐linked PF4 molecules reactive with either VITT or HIT Abs. Using these antigenic targets in the same diagnostic platform allows us to distinguish the presence of VITT or HIT Abs in a given serum sample. Labeling electrodes with gold nanoparticles carrying anti‐human IgG and Pb^2^⁺ (AuNPs/anti‐human IgG@Pb^2^⁺) is followed by voltammetry to produce electrochemical signals proportional to the concentration of surface bound anti‐PF4 Abs. B) Electrodes carrying biorecognition molecules are incorporated into a microfluidic device. The device (microfluidic electrochemical biosensor) contained multiple microchambers to enable parallel analysis of patient sera as well as positive and negative controls. Microvalve integration in the device allowed full automation of labeling‐incubation‐washing cycle while using only 15 µL of diluted patient sera.

In this study, we sought to combine an electrochemical immunoassay with microfluidic automation to develop a rapid, sensitive, and specific assay for detecting and differentiating HIT/HIT‐like and VITT/VITT‐like Abs. Initial preliminary studies suggested that cross‐linked PF4 (c‐PF4) molecules represent a specific antigenic target specific for VITT‐like Abs, while PF4 molecules complexed with heparin are bound by both HIT/HIT‐like and VITT/VITT‐like Abs. Here, this observation was leveraged to design a sensing strategy shown in **Figure** **1**A, where Au electrodes were functionalized with heparin/PF4 complexes, incubated with patient sera, and then labeled with immunoprobes. Binding of VITT/VITT‐like or HIT/HIT‐like Abs to the antigenic target was determined electrochemically. Importantly, sensing electrodes were incorporated into a series of valve‐controlled microfluidic chambers and were used to determine VITT vs HIT status based on an input of a small volume of serum, 15 µL, in a fully automated fashion (see Figure 1B).

## 2 Results

### 2.1 Modifying PF4 to Tune Antigenic Specificity to VITT Abs

**Figure 2:**
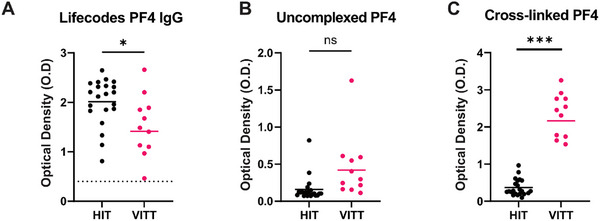
Designing antigenic target to improve specificity for VITT Abs. A cohort of patients with clinically confirmed HIT (n = 21) or VITT (n = 11) were tested with ELISAs utilizing A) PF4/polyanion, B) uncomplexed PF4, or C) cross‐linked PF4 targets, to compare the sensitivity and specificity of each antigenic target for detecting HIT or VITT anti‐PF4 antibodies among a cohort of patient samples.

A key goal of this study was to develop antigenic targets for VITT/VITT‐like Abs to be able to differentiate them from closely related HIT/HIT‐like Abs. PF4 is the antigenic target for both antibody (Ab) types (see Figure S1A, Supporting Information, for amino acid sequence). However, previous studies suggest that each Ab type recognizes different epitopes.^[^ ^40^ ^]^ Previously, it has been established that HIT Abs recognize PF4 complexed to heparin or other polyanions^[^ ^41^ ^]^; and as expected, a commercial ELISA utilizing PF4/polyvinyl sulfonate complexes did not allow for the discrimination of HIT and VITT antibodies (**Figure** **2**A). Our team has previously shown that VITT Abs interact with uncomplexed PF4 and that uncomplexed PF4 may be used in ELISA for VITT detection.^[^ ^18^ ^]^ However, this past study was carried out with a cohort of only six VITT patients. When a larger cohort of samples (11 VITT and 21 HIT), including the six VITT samples previously analyzed, were tested with ELISA using uncomplexed PF4 as antigenic target, we did not observe satisfactory differentiation between the two groups (Figure 2B). We note that the OD values obtained in the current study with uncomplexed PF4 were lower than those reported previously, a result confirmed even after retesting with additional PF4 lots. We hypothesize that the specific lot of the PF4 preparation used in the earlier study may have contained higher order complexes, which would account for the previous, higher signal. These findings underscore the inherent variability of using uncomplexed PF4 as an antigen for VITT detection. To overcome this limitation and establish a more specific assay, we explored cross‐linking uncomplexed PF4 as a more specific antigen target for VITT Abs. Pursuit of this approach was catalyzed by our observation that gentle cross‐linking of PF4 molecules with 1‐ethyl‐3‐(3‐dimethylaminopropyl) carbodiimide (EDC) provided high‐specificity for VITT Ab binding (Figure S1A, Supporting Information). Notably, optimal cross‐linking conditions did not significantly alter PF4 oligomerization. As shown by Sodium Dodecyl Sulfate‐Polyacrylamide Gel Electrophoresis (SDS‐PAGE) and ultracentrifugation (Figure S1C,D, Supporting Information), cross‐linking of PF4 yielded a mixture of oligomeric species, with ≈81.2% of PF4 present as tetramers.

Importantly, when cross‐linked (c)‐PF4 was used as antigenic target in an ELISA assay, VITT sera samples produced significantly higher signals compared to HIT sera (Figure 2C). This supported our hypothesis that cross‐linking may change the conformation of PF4 to improve specificity for VITT Abs, and studies to improve the mechanisms of how the process of cross‐linking exposes epitopes and improves VITT Abs binding is ongoing. In the meantime, we wanted to leverage this exciting observation toward constructing a VITT diagnostic assay suitable for deployment in the frontline or point‐of‐care setting.

### 2.2 Design of the Electrode Interface to Enable Sensitive and Specific Detection of HIT and VITT

**Figure 3:**
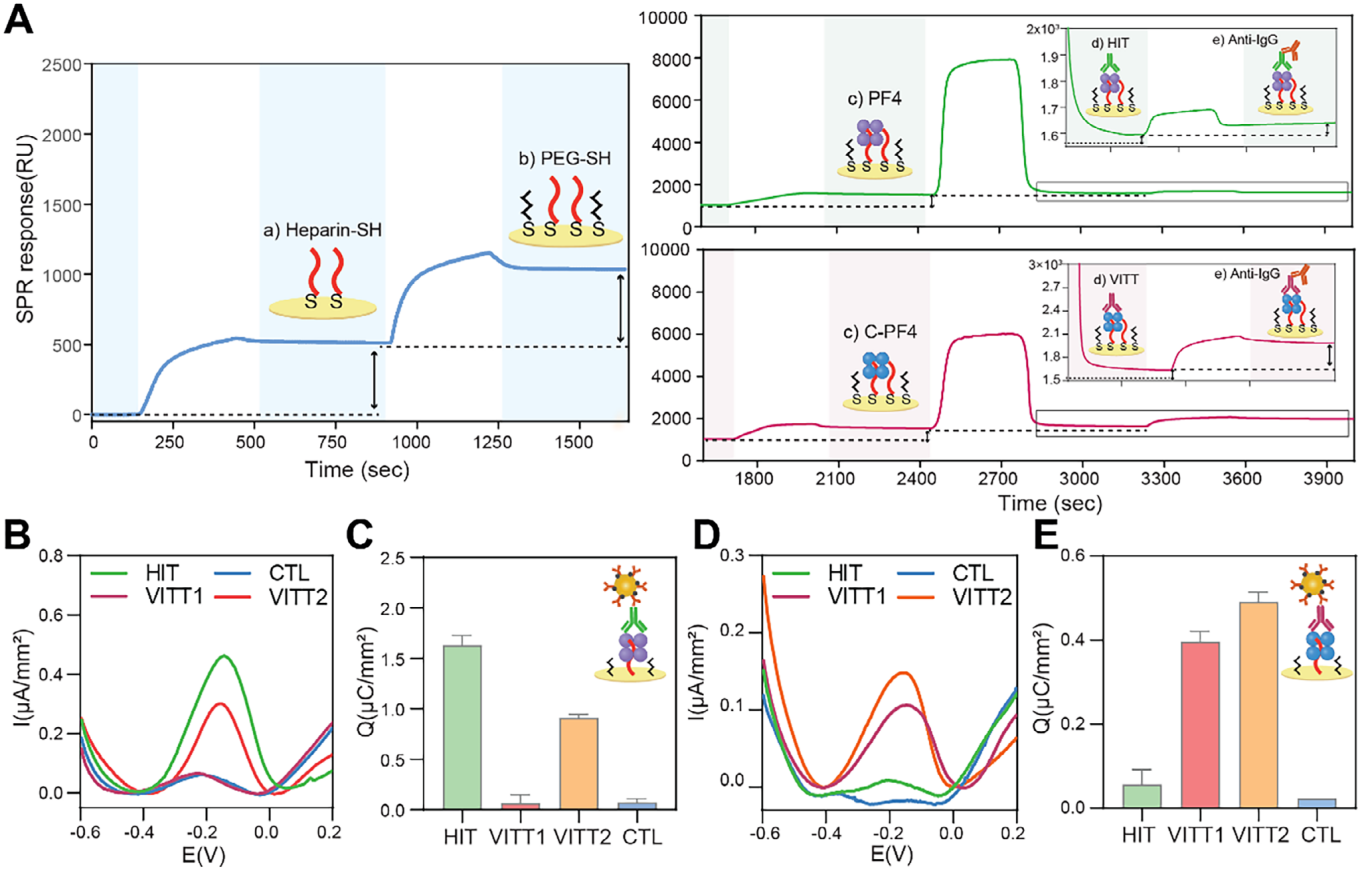
Assembly of the biorecognition layer and analysis of biosensor specificity toward HIT or VITT Abs. A) SPR analysis of steps involved in the assembly of the biorecognition layer: a) Immobilization of heparin‐SH, b) passivation with PEG‐SH, and c) binding of either PF4 or c‐PF. PF4‐containing biorecognition layer was challenged with HIT serum while c‐PF4 layer was exposed to VITT serum. Anti‐human IgG Abs were used enhance the signal. B) SWV signals from electrodes functionalized with PF4 and exposed to either healthy serum (blue), HIT serum (green), VITT1(red), or VITT2 (orange) serum. This result illustrates that HIT sera produced higher signals than control but that some VITT samples (e.g., VITT2) produced high signals as well. Thus, the ability to discriminate HIT from VITT was limited when using PF4 as antigenic target. C) Normalized charge values for experiment described in part (B). D) SWV signals for electrodes functionalized with c‐PF4 and challenged with healthy (blue), HIT (green), VITT1 (red), or VITT2 (orange) patient sera. Data in panels (C) and (E) are shown as mean ± SD. This result demonstrated specificity for VITT Abs. E) Normalized charge values for experiment described in part (D).

Building on the observation that cross‐linking of PF4 improves immunoassay sensitivity toward VITT Abs, we proceeded with the development of electrochemical immunoassays. Given that heparin is commonly complexed with PF4 for detecting anti‐PF4 Abs, we wanted to explore a strategy for immobilizing heparin/PF4 complexes on an electrode surface. To achieve this, heparin was functionalized to contain thiol groups because thiolated molecules form strong, stable bonds with Au substrates. Subsequently, surface plasmon resonance (SPR) was used to characterize and optimize the assembly of the biorecognition layer comprised of heparin‐thiol (Hep‐SH) and either PF4 or c‐PF4. The use of SPR allowed us to optimize construction of the biorecognition layer on the Au substrate and apply the optimized protocol for electrochemical microfluidic biosensing experiments described later in the paper. **Figure** **3**A shows a representative SPR sensogram depicting Hep‐SH binding and poly(ethylene glycol)‐thiol (PEG‐SH) blocking steps. After creating the Hep‐SH/PEG‐SH layer, the SPR chips were exposed to either PF4 or c‐PF4 to create a complex for recognizing HIT or VITT Abs. These surfaces were then incubated with HIT or VITT patient serum. As a final step, anti‐human imunoglobulin g (IgG) Abs were introduced into the SPR flow cells to confirm the presence of human IgG Abs on the surface. Given that serum contains multiple proteins capable of interacting with heparin^[^ ^42^ ^]^, labeling with anti‐human IgG Abs was used to reveal the presence of IgG Abs rather than non‐specific serum protein interactions. Additionally, although PF4 binding was comparable at 0.25 and 0.5 mg mL^−1^ Hep‐SH (Figure S2C, Supporting Information), the surface prepared with 0.25 mg mL^−1^ provided a higher HIT/CTL response ratio after serum and anti‐IgG binding (Figure S2e, Supporting Information), due to reduced nonspecific interactions of control (CTL) serum and more efficient PEG blocking. SPR results confirmed the successful construction of a biorecognition layer comprised of Hep‐SH/PF4 or Hep‐SH/C‐PF4 complexes and the capture of Abs from HIT or VITT serum followed by the binding of anti‐human IgG to the HIT and VITT Abs (Figure 3A,B).

Having characterized the construction of the biorecognition layer and binding of HIT or VITT Abs to this layer, we designed the electrochemical biosensor integrated into a simplified microfluidic device. As shown in Figure S3 (Supporting Information), this device was fabricated in poly(dimethyl siloxane) (PDMS). It contained six independent microfluidic compartments, each with a microchamber of 1 µL and two Au working electrodes (600 µm diameter) patterned on glass. The electrodes were functionalized using a sequence of steps outlined in Figure 3A. However, unlike SPR analysis where anti‐human IgG Ab molecules were used, electrochemical detection was carried out with immunoprobes (AuNPs/anti‐human IgG@Pb^2^⁺). As shown in Figure S3A (Supporting Information), square wave voltammetry (SWV) analysis initially revealed a high background signal in the control sample. The total charge (Q) was calculated from the SWV curves, and the signal‐to‐noise ratio (Q_HIT_/Q_CTL_) was found to be 2.6 (Figure S4B, Supporting Information). To determine whether the signal‐to‐noise ratio could be improved through better passivation of the electrodes, we evaluated experimental parameters that included three different passivation agents and various incubation times. These experiments revealed that the suboptimal S/N was attributable to the non‐specific interactions between the electrode and the immunoprobes (Figures S5 and S6, Supporting Information). Passivating the electrodes with PEG‐SH for 1 h minimized non‐specific binding of immunoprobes as confirmed by SPR (see Figures S6 and S7, Supporting Information) and allowed us to increase S/N of electrochemical detection to 8.8 for the same serum sample that previously yielded an S/N of 2.8 (Figure S3, Supporting Information). Next, we evaluated the specificity of the electrodes functionalized with PF4 and c‐PF4. Electrodes containing either target antigen were challenged with control, HIT, or VITT serum. Figure 3B–E summarizes the electrochemical responses for the different antigen/serum combinations. These results confirm that our electrochemical biosensor was specific to either HIT or VITT Abs depending on the antigen used.

### 2.3 Design and Operation of the Automated Microfluidic Electrochemical Biosensor

**Figure 4:**
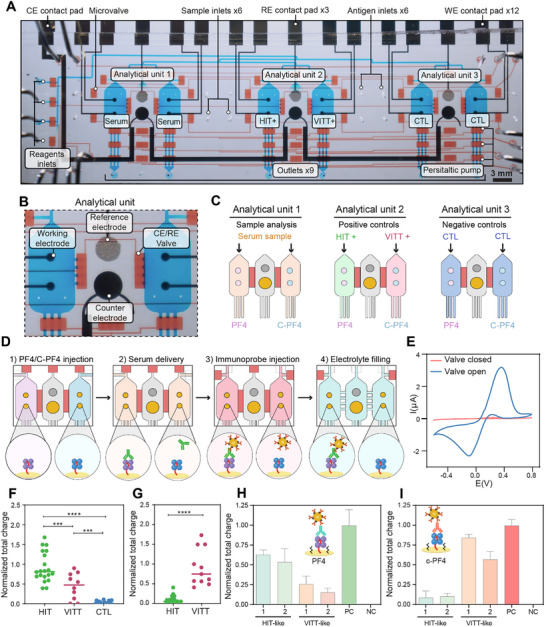
Automated operation of the microfluidic device and analysis of patient sera. A) Photograph highlighting device components where the valve (control) layer filled was filled with blue dye and the flow layer was filled with red dye. B) Close‐up view of one analytical unit comprised of three microchambers. Two microchambers contained working electrodes and were flanking a chamber with reference and counter electrodes. Fluidic connection between the chambers was controlled by microvalves. C) Description of how analytical units were used in an assay. D) Illustration showing steps of the assay: 1) immobilization of antigen targets, 2) introduction of serum samples, 3) labeling with immunoprobes, and 4) actuation of microvalves to connect working electrodes to counter and reference electrodes for electrochemical measurements. E) [Fe(CN)6]^4‐/3−^ cyclic voltammetry demonstrating that the connection to counter and reference electrodes was established only with opened microvalves. F) Analysis of patient sera samples in a microfluidic electrochemical biosensor with PF4 as antigenic target. HIT samples (n = 20) produced significantly higher signals than VITT samples (n = 11) and controls (n = 20). G) Analysis of patient sera in a microfluidic biosensor with c‐PF4 as antigenic target. VITT samples (n = 11) produced signals that were significantly higher compared to HIT sera (n = 20). Each dot represents the mean of two technical replicates. Statistical significance was determined using a two‐tailed Mann–Whitney U test (*p* < 0.05; ^**^ *p* < 0.001; ^***^ *p* < 0.0001). H,I) Normalized charge values of two HIT‐like and two VITT‐like anti‐PF4 sera samples on PF4 (H) and c‐PF4 (I) are shown. The patients with HIT‐like anti‐PF4 sera were previously described in refs. [9, 42, 43] VITT‐like anti‐PF4 sera were previously described in refs. [10, 43, 44] (Patient 1). Positive controls (PC) correspond to recombinant HIT‐like (for PF4) and VITT‐like (for c‐PF4) antibodies.

Given the ultimate goal of eliminating manual handling, we proceeded to automate the assay workflow in the microfluidic device. The design criteria for this device were 1) to enable the detection of both HIT/HIT‐like and VITT/VITT‐like, 2) to incorporate positive and negative controls for signal normalization, and 3) to utilize a minimal volume of serum for analysis. **Figure** **4**A,B shows the device developed to accomplish these goals. The device contained three analytical units, each comprising two sensing compartments and one compartment housing reference (RE) and counter electrodes (CE). The device also contained a series of pneumatic microvalves used for liquid routing and pumping. Figure 4B offers a close‐up view of one sensing unit. As may be noted, each sensing compartment contained two working electrodes. Figure 4C illustrates the configuration of the three analytical units, where each sensing compartment contains either PF4 or c‐PF4. The first unit was designed for serum sample analysis, the second for positive controls specific to HIT/HIT‐like and VITT/VITT‐like, and the third for negative controls (CTL). The sensing and RE/CE compartments were connected by pneumatically controlled microvalves that were kept closed to isolate working electrodes during serum incubation and immunoprobe labeling steps and were opened only when making electrochemical measurements. This allowed us to keep the RE/CE compartment pristine and unaffected by serum before electrochemical measurements.

The automated assay workflow of one of the three units is shown in Figure 4D and Movie S1 (Supporting Information). It starts by isolating the CE/RE compartments from sensing compartments. The biorecognition layer was constructed on the working electrodes using either PF4 or c‐PF4. Next, a test serum sample as well as positive and negative controls are pumped into the sensing compartments for 10 min (Movie S2, Supporting Information). On‐board peristaltic pumps allow us to use as little as 15 µL of patient sample during testing. After washing with Phosphate‐Buffered Saline‐Tween (PBS‐T), immunoprobes were flowed into all the chambers for 10 min and then washed again with PBS‐T and DI water. Subsequently, an electrolyte (acetic acid/sodium acetate buffer) is introduced into all compartments, and the valves isolating the sensing from CE/RE compartments were opened. Figure 4E describes [Fe(CN)_6_]⁴^−^/^3^^−^ cyclic voltammetry performed in conjunction with valve actuation and shows that the electrochemical signal was only observed when channels connecting working electrodes to CE/RE electrodes were opened.

### 2.4 Performance of the Microfluidic Automated Electrochemical Biosensor

Before testing serum samples, we carried out extensive characterization of the device. First, we evaluated the electrochemical performance of each of the 12 working electrodes in the device to confirm that the location of the electrodes did not affect their properties. Figure S8A,B (Supporting Information) shows representative cyclic voltammetry and SWV responses of electrodes in 10 mm [Fe(CN)6]^4‐/3−^ in 0.1 m KCl. As may be appreciated from these data, anodic and cathodic peak currents were similar between the working electrodes regardless of location, with a coefficient of variation (*CV*) of less than 5%.

We next evaluated biosensor responses to HIT‐like and VITT‐like Abs identified in two patients diagnosed with HIT‐like and VITT‐like monoclonal gammopathy of thrombotic significance (MGTS), respectively, whose monoclonal anti‐PF4 Abs were de novo sequenced and produced recombinantly. These Abs provided us with a positive control for HIT detection and were used to establish analytical performance (limit of detection, dynamic range) of our electrochemical biosensor. Challenging electrodes with varying concentrations of HIT‐like Abs allowed us to establish a limit of detection (0.12 µg mL^−1^) and dynamic range (1.3 µg mL^−1^) of the electrochemical biosensor (Figure S9A,B, Supporting Information). Similarly, biosensor responses to VITT‐like recombinant Abs were evaluated using c‐PF4‐functionalized electrodes, yielding a limit of detection of 2.9 µg mL^−1^ and a dynamic range extending up to 48 µg mL^−1^ (Figure S9C,D, Supporting Information).

### 2.5 Validation of Microfluidic Automated Electrochemical Biosensor Using a Cohort of VITT and HIT Samples

Our microfluidic automated electrochemical biosensor was validated using a cohort of 51 serum samples. Twenty samples were from patients who tested positive for HIT Abs using commercial assays, eleven were from patients whose VITT diagnosis was confirmed by platelet function assay, while the remaining twenty serum samples were from healthy controls. Demographic, laboratory, and clinical information for both VITT and HIT patient cohorts is provided in Tables S1 and S2 (Supporting Information), including patient age, sex, platelet counts, thrombosis sites, and anti‐PF4 ELISA optical density values. Additional parameters, such as vaccine type and time from vaccination for VITT patients and cardiac surgery history for HIT patients, are also included.

Electrochemical signals were recorded from two different working electrodes, and the total charge (Q) was calculated by integrating the area under the SWV curve. The normalized charge values (Q̅) were calculated using the formula (Q_S_‐Q_NC_)/(Q_PC_‐Q_NC_), where Q_S_ represents the charge of the clinical serum sample, Q_NC_ represents the charge of the negative control (control serum), and Q_PC_ represents the charge of the positive control (HIT‐like Abs or VITT positive serum sample). As shown in Figure 4F, electrodes functionalized with PF4/heparin complexes showed significantly higher signals in HIT sera compared to healthy sera, as well as a significant difference between HIT and VITT sera and between VITT and control sera. These findings indicate that PF4/heparin provides some level of differentiation between HIT and VITT, although with overlapping signals in some VITT samples. This result was unsurprising given that other immunoassays relying on PF4‐polyanion complexes as antigenic targets have shown a limited ability to distinguish HIT and VITT Abs.^[^ ^18^ ^]^ However, specific detection of VITT sera was achieved using electrodes containing c‐PF4/heparin complexes. As shown in Figure 4G, VITT sera produced significantly higher signals compared to HIT sera. These results are exciting as they demonstrate, for the first time, the ability to differentiate between HIT and VITT sera samples in the same diagnostic platform. Figure 4H,I further demonstrates that the device can differentiate VITT‐like anti‐PF4 antibodies from HIT‐like anti‐PF4 antibodies outside the context of VITT and HIT, with recombinant HIT‐like and VITT‐like monoclonal antibodies serving as positive controls. These disorders are being increasingly recognized as causes of unexplained thrombosis.^[^ ^9^, ^10^, ^22^, ^43^, ^44^ ^]^

These data suggest that the use of c‐PF4 significantly enhances specificity for detecting VITT‐like Abs, making it a more specific and similarly sensitive alternative to HIT ELISA testing for detecting VITT‐like antibodies. In addition, our data show that the use of uncross‐linked PF4 functionalized heparin electrodes is highly accurate for detection of HIT antibodies.

## 3 Discussion

Our study addresses the critical need for technically simple, rapid assays capable of detecting VITT‐like antibodies, particularly ones that can be performed outside of specialized clinical laboratories. It describes the development of an automated microfluidic electrochemical biosensor designed for the detection of HIT/HIT‐like and VITT/VITT‐like Abs using only 15 µL of serum. Specificity of the biosensor is ensured by a novel biorecognition layer containing either Heparin‐SH/PF4 or Heparin‐SH/c‐PF4 complexes. These biorecognition molecules are assembled on miniature electrodes and integrated into a microfluidic device. The device also incorporates computer‐controlled microvalves for routing and pumping microliter volumes in a fully automated manner. The microfluidic electrochemical biosensor included multiple microchambers to parallelize testing of serum samples as well as positive and negative controls. The current device with integrated pneumatic microvalves is fabricated in PDMS—a material well‐suited for prototyping and testing in the lab but less amenable to translation and commercialization. However, there have been recent successful efforts to implement pneumatic microvalves in thermoplastic devices fabricated by molding or milling.^[^ ^36^, ^39^ ^]^ Therefore, we do not envision difficulties fabricating a device with similar level of automation using thermoplastic materials amenable to scalable manufacturing. Similarly, while control of pneumatic microvalves is typically accomplished with bulky pneumatic system in a research lab setting, there are multiple reports of portable control systems incorporating low‐cost electronics.^[^ ^45^ ^]^ These advances highlight the path to translating microfluidic devices with integrated pneumatic microvalves into cost‐effective and portable point‐of‐care systems.

Beyond its technical benefits, our biosensor has high clinical significance. Traditionally, thrombotic disorders associated with anti‐PF4 Abs have been connected to heparin‐based anticoagulation in the context of HIT.^[^ ^46^, ^47^ ^]^ However, recent research suggests that patients can also produce platelet‐activating anti‐PF4 Abs in several life‐threatening scenarios that occur independently of heparin exposure.^[^ ^4^, ^9^, ^22^ ^]^ VITT is one such disorder that emerged following the administration of adenoviral COVID‐19 vaccines.^[^ ^1^, ^2^, ^3^ ^]^ Distinguishing VITT from HIT remains challenging, particularly because both conditions involve anti‐PF4 antibodies with overlapping antigenic profiles.^[^ ^21^ ^]^ Because of this, a definitive diagnosis of VITT necessitates meeting several criteria: a documented history of recent vaccination, thrombocytopenia and/or thrombosis, and the presence of a demonstrated platelet activating antibody based on confirmatory functional platelet assays.^[^ ^7^, ^9^ ^]^ Recent efforts to develop VITT‐specific assays include a chemiluminescence‐based test that uses PF4 covalently cross‐linked to magnetic beads.^[^ ^19^ ^]^ This assay demonstrates high specificity for VITT Abs, however, its implementation requires access to specialized instrumentation (e.g., the ACL AcuStar platform), which limits its availability and point‐of‐care applicability. Additional efforts to differentiate HIT and VITT antibodies have been performed using competitive inhibtion of antibody binding in anti‐PF4 ELISA using a monoclonal antibody (1E12).^[^ ^20^ ^]^

While the incidence of VITT is low (3–15 cases per million vaccinations),^[^ ^2^ ^]^ our biosensor may have significance beyond detecting VITT Abs. An important recent development in the field of thrombotic disorders is the identification of thrombosis‐causing VITT‐like and HIT‐like Abs in individuals without prior vaccination or exposure to heparin in a condition called monoclonal gammopathy of thrombotic significance (MGTS).^[^ ^9^, ^10^, ^22^, ^43^, ^44^ ^]^ Although quite rare, MGTS patients represent are subset of a much larger cohort of individuals (3% of US adult population) who present with monoclonal gammopathies of undetermined significance (MGUS). Our technology may be used in the future to identify HIT‐ and VITT‐like antibodies in MGTS or MGUS patient cohorts. There is also emerging evidence of patients with VITT‐like anti‐PF4 antibodies in the absence of MGUS.^[^ ^44^ ^]^ Therefore, ability to detect VITT or VITT‐like antibodies afforded by our electrochemical biosensor may have utility across a number of clinical conditions.

We should note that currently VITT diagnosis is made by evaluating a panel of clinical criteria, including history of recent vaccination, platelet count, positive ELISA result for HIT, and PF4‐dependent functional assays. Our microfluidic electrochemical biosensor represents an improvement on the existing ELISA approaches and is intended for use in combination with other clinical criteria. It is also worth noting that additional studies will be needed to validate our biosensor against patient cohorts with complex forms of HIT and other thrombotic disorders. These future studies will establish specificity of this biosensor and fully characterize its positive predictive value in different clinical settings.

## 4 Experimental Section

### Study Design

This study was designed to address the need for sensitive detection and differentiation of VITT and HIT antibodies. The study design was based on the following criteria: 1) the development of specific and sensitive assays using two biorecognition layers with target antigens for each thrombotic condition and 2) the integration of a nanoparticle‐enabled electrochemical immunoassay into an automated microfluidic device to eliminate manual handling and reduce serum volume requirements for limited sample availability, particularly for VITT. Serum samples from VITT patients, HIT patients, and healthy controls were collected retrospectively. HIT serum samples were from HIT‐suspected patients who tested positive through Lifecodes PF4 IgG HIT ELISA and platelet‐based functional assays (Serotonin Release Assay or the PF4‐dependent P‐Selectin Expression Assay) in the Mayo Clinic Special Coagulation Laboratory. VITT serum samples were collected from VITT‐confirmed cases based on the clinical history and laboratory confirmation (Lifecodes PF4 IgG HIT ELISA and PF4‐dependent P‐Selectin Expression Assay). Control samples were obtained from healthy individuals. Blood samples were obtained with consent from patients suspected of MGTS and used in research testing. Studies were approved by the Institutional Review Board of Mayo Clinic (IRB# 20–001608).

### Materials

4‐(2‐Hydroxyethyl)piperazine‐1‐ethanesulfonic acid (HEPES), Pb(NO3)2, Ammonium acetate, Tween 20, chlorotrimethylsilane, Amicon Ultra‐0.5 Centrifugal Filter, bovine serum albumin, and 6‐Mercapto‐1‐hexanol, sodium chloride (NaCl), 1‐ethyl‐3‐(3‐dimethylaminopropyl) carbodiimide (EDC), cysteamine, and dithiothreitol (DTT) were purchased from Sigma–Aldrich (St. Louis, MO, USA). Hydroxy‐EG6‐undecanethiol was obtained from Dojindo Molecular Technology (Washington, DC, USA). Sodium heparin (Mw: 12 kDa) was purchased from Smithfield Bioscience (Cincinnati, OH, USA). 4‐Arm poly (ethylene glycol) thiol (PEG4SH; Mw: 10 kDa) was obtained from Sunbio Inc. (Anyang, Korea). 1 – Hydroxybenzotriazole hydrate (HOBt) was purchased from Anaspec (Fremont, CA, USA). A dialysis membrane was obtained from Spectrum Laboratories (3.5 kDa Mw cut‐off, Rancho Dominguez, CA, USA). NHS‐activated AuNPs (20 nm) were purchased from CytoDiagnostics (Tulsa, OK, USA). Ethyl alcohol (EtOH) was purchased from Electron Microscopy Sciences (Hat eld, PA, USA) and isopropyl alcohol (IPA) was acquired from Honeywell (Charlotte, NC, USA). Platelet‐factor 4 (Human CXCL4) was purchased from Protein Foundry LLC (Milwaukee, WI, USA). Human IgG Antibody was purchased from R&D Systems (Minneapolis, MN, USA). SuperBlock Blocking Buffer and phosphate‐buffered saline (PBS) were obtained from Thermo Fischer Scientific (Waltham, MA, USA). Silicon wafers were purchased from University Wafer (Boston, MA, USA). SU‐8 2025 photoresists and SU‐8 developer were acquired from Kayaku Advanced Materials (Westborough, MA, USA). AZ 5214‐E IR photoresist, AZ 300 MIF developer, AZ 10XT photoresist, and AZ‐400K developer were purchased from Integrated Micro Materials (Argyle, TX, USA). Poly(dimethylsiloxane) (PDMS) base and curing agent kit (Sylgard 184) were obtained from Ellsworth Adhesives (Minneapolis, MN, USA). Gold etch‐type TFA was purchased from Transene Electronic Chemicals (Danvers, MA, USA). CR‐7s Chrome Etch was purchased from KMG Electronic Chemicals (Pueblo, CO, USA). Ag/AgCl ink was acquired from CH Instruments (Bee Cave, TX, USA). 3‐(3‐dimethylaminopropyl) Carbodiimide (EDC), 4‐Morpholineethanesulfonic acid (MES), and TRIS buffer were purchased from Millepore Sigma (Burlington, MA, USA).

### Fabrication of Electrode Arrays

Glass slides were used as substrates for electrode array fabrication. A 10 nm Cr adhesion layer and a 100 nm Au layer were sputter‐coated onto the glass slides (Lance Goddard Associates, Santa Clara, CA). Next, a 1 µm layer of AZ 5214‐E IR photoresist was spin‐coated onto the substrate at 5000 rpm and soft baked at 110 °C for 1 min. The desired electrode structures were UV exposure using a micropattern generator (µPG 101, Heidelberg Instruments, Germany), and the exposed photoresist was removed using 300 MIF developer. Subsequently, the patterned Au/Cr layers were etched to create electrodes. To remove any remaining unexposed photoresist, the electrode‐patterned slide was sonicated in acetone and then with IPA. Additionally, oxygen plasma treatment was applied for 2 min to ensure surface cleanliness. To construct the on‐chip Ag/AgCl reference electrodes, Ag/AgCl ink was applied onto the Au electrode surface and cured at 120 °C for 20 min.

### Fabrication of Molds

The microfluidic devices were designed using CAD software and fabricated using photolithography and soft lithography techniques. Initially, three master molds were fabricated: one for the simple device and two for the automated microfluidic device (a control and a flow layer mold). The simple device mold and the control layer mold of the automated device were fabricated using the same protocol. First, two 4″ silicon wafers were spin‐coated with negative photoresist (SU8 2025) at 1300 rpm to achieve a 55 µm layer thickness, followed by soft baking on a hot plate at 65 °C for 3 min and 95 °C for 9 min. Subsequently, the photoresists were exposed to UV laser using a micropattern generator (µPG‐101, Heidelberg Instruments Inc.). The molds were then baked on a hot plate at 65 °C for 2 min and 95 °C for 7 min, developed by immersion in SU8 developer, and hard baking at 150 °C for 30 min.

To fabricate the flow layer of the automated microfluidic device, a silicon wafer was first coated with a 30 µm‐thick layer. First, the wafer was spin‐coated with two consecutive layers of positive AZ10XT photoresist at 800 rpm with soft bakes of 80 s at 110 °C and 180 s at 115 °C. The structures were then UV‐exposed and developed with AZ 400k 1:3 developer. A reflow process was performed by baking the mold at 120 °C for 30 min to obtain rounded structures. Then, the temperature was increased to 200 °C with a ramp of 3 °C min^−1^ and hard baked for 120 min. Subsequently, the mold was coated with a negative SU8 2025 photoresist layer using the same protocol for the control layer. Finally, all three molds were exposed to chlorotrimethylsilane for 1 h.

### Fabrication of PDMS Devices

The PDMS base and curing agent were mixed to fabricate the simple devices at a 10:1 ratio (w/w). This mixture was poured into the mold, degassed in a vacuum desiccator for 10 min, and baked in a convection oven for 1 h at 80 °C. The cross‐linked PDMS was then peeled off from the mold, and the inlets and outlets were punched using a 0.4 µm puncher. Finally, the PDMS devices were treated with oxygen plasma for 2 min and aligned onto the array of electrodes.

Automated microfluidic device replicas were fabricated by pouring the PDMS base and curing agent at a 5:1 (w/w) ratio onto the control layer mold and placing them in a vacuum desiccator for 10 min. The flow layer mold was spin‐coated at 600 rpm with a PDMS‐curing agent mix at a 20:1 ratio. Subsequently, both molds were partially cured at 80 °C for 20 min. Afterward, the control layer replicas were peeled off, cut, and aligned onto the flow layer mold. Both layers were baked together for 90 min at 80 °C to bond them. Finally, the multilayer devices were peeled off, and the inlets and outlets were punched before bonding the replicas onto the array of electrodes using oxygen plasma treatment.

### Preparation of the AuNPs/Anti‐Human IgG@Pb2+ Immunoprobes

We have previously described a protocol to conjugate AuNPs/Abs@Pb^2+^.^[^ ^22^ ^]^ We followed this protocol for this study to synthesize immunoprobes targeting human IgG (AuNPs/anti‐human IgG@Pb2+). Briefly, NHS‐activated AuNPs (6.54 × 1011 NPs mL^−1^) were dispersed in 90 µL of 1× PBS containing 40 µg of antibody (Ab) solution and incubated for 4 h at room temperature. Thereafter, 10 µL of quencher solution was added to eliminate the remaining unreacted NHS on AuNPs and then incubated with BSA to block the AuNP.

To remove unbound Abs and any reagents, the resulting mixture was centrifuged at 7000 × *g* for 20 min and washed three times with ammonium acetate buffer (20 mm, pH 6) with 0.05% Tween 20 Next, the synthesized AuNPs/Abs conjugates were dispersed in 0.15 mL of ammonium acetate buffer with 0.025% Tween 20, and incubated with 15 µL of 10 mm Pb(NO3)_2_ of aqueous solution and kept stirring overnight to complex Pb^2+^ ions with the amine group of Abs. Finally, the AuNPs/Abs@Pb^2+^ were collected by centrifugation and washed thoroughly with 1 mL of HEPES buffer (0.02 m, pH 7.0) with 0.025% Tween 20.

### Synthesis of Heparin‐Thiol

Thiolated heparin (Hep‐SH) was synthesized by converting the carboxyl groups of heparin into thiol groups, as described in a previous report.^[^ ^48^ ^]^ Hep‐SH with a 30% degree of thiolation was prepared by reacting the carboxyl groups of heparin with cysteamine. Briefly, heparin was dissolved in distilled water (DW) at a concentration of 10 mg mL^−1^. EDC (1.5 molar ratio), HOBT (1.5 molar ratio), and cysteamine (2 molar ratio) were sequentially added, and the pH was adjusted to 6.8. After reaction under stirring for 12 h at room temperature, the solution was thoroughly dialyzed to remove unreacted reagents. Then, an excess amount of DTT (10 molar ratio) was added to reduce the oxidized disulfide groups and to form free thiol groups on the modified heparin. After 12 h of reaction, this solution was dialyzed against a 0.1 m NaCl solution at pH 3.5, then at pH 5.0, and finally in pure DIW, sequentially, and then lyophilized. The degree of thiolation was measured using Ellman's reagent at 412 nm. 30% thiolated heparin was used for all experiments.

### Device Setup and Operation

First, the microvalves were filled with DI water using Tygon tubing connected to the pneumatic control system delivering a 30‐psi pressure. The pneumatic control panel comprised solenoid valves (MH1, Festo), which were controlled with a microcontroller (MEGA 2560, Arduino) via a LabVIEW interface (2023 community edition). The flow layer channels were filled with PBS‐T. Afterward, the microvalves were actuated to isolate the reaction chamber form the central chamber containing the onboard reference/counter electrode. Next, to functionalize the working electrode surfaces, Heparin‐SH, PEG‐SH, PF4, cross‐linked PF4, and PBS‐T were loaded into the device, and the automated program to surface preparation was activated. To functionalize the working electrode surfaces, the serum samples, anti‐human IgG@Pb2+ nanoparticles, and the electrolyte were loaded, and the assay was automatically performed by activating the LabVIEW program. The device uses on‐chip peristaltic pumps to draw the reagents and samples into the reaction chambers. The pumps operate at a frequency of 100 ms, achieving a volumetric flow rate of 1 µL min^−1^ with a total assay runtime of 50 min.

### Functionalization of Electrodes and Electrochemical Detection of HIT and VITT Antibodies

The Au working electrodes were functionalized with the antigen targets PF4 or cross‐linked PF4 to capture HIT or VITT antibodies. The preparation of the biosensor surface involves initially functionalizing the working electrodes with Heparin‐thiol (0.25 mg mL^−1^) by flowing for 10 min, followed by washing with phosphate‐buffered saline with 0.05% Tween 20 (PBS‐T) for 10 min. Subsequently, working electrodes are blocked with PEG‐thiol (1 mg mL^−1^) by injecting for 10 min and then incubating for 1 h. After washing, PF4 or cross‐linked PF4 (20 µg mL^−1^) was immobilized on the working electrodes. The functionalized electrodes are exposed to serum samples for 10 min to detect HIT and VITT antibodies, followed by washing with PBS‐T. Next, the anti‐human IgG@Pb2+ nanoparticles were injected at a concentration of 6.54E^+11^ particles/mL for 10 min. After washing the electrodes with PBS‐T and DI water, microvalves to isolate the central chamber were opened to establish an electrolyte connection between the working electrode and working, counter, and reference electrodes. Finally, the device was connected to a potentiostat (PalmSens) and analyzed using square wave voltammetry. The SWV curves were recorded in the electrolyte (acetic acid/sodium acetate buffer; 0.2 m, pH 4.5) in the −0.9–0.1 V range (vs Ag/AgCl) with 25 mV amplitude and 15 Hz frequency.

### Surface Plasmon Resonance Experiments

A surface plasmon resonance (SPR) system (Biosensing Instruments) was used to confirm surface functionalization steps and to optimize the biosensor surfaces. Au SPR chips were prepared by flowing Heparin‐SH (0.25 mg mL^−1^), PEG‐SH (1 mg mL^−1^), PF4 (20 µg mL^−1^), serum samples (1:50 dilution in super blocking buffer), and anti‐human IgG antibodies (1:100 dilution in PBS). For the optimization of non‐specific binding of anti‐human IgG@Pb^2+^ gold nanoparticles, Au SPR chips were incubated for 1 h with mercaptohexanol (MCH), polyethylene glycol‐thiol (PEG‐SH), or hydroxy‐EG6‐undecanethiol. Then, the anti‐human IgG@Pb^2+^ nanoparticles were injected at 20 µL min^−1^. Resonance units (RU) were acquired from the baseline changes before and after the reagent injection.

### Chemical Cross‐Linking of PF4 Polypeptides

For PF4 cross‐linking, 1 mg mL^−1^ EDC and recombinant PF4 were added at an equal ratio (v:v) in 15 mm MES buffer pH 8.0 for 6 h at ambient temperature. Cross‐linking reactions were stopped by adding 1 m Tris pH 7.4 buffer (i.e., a primary amine‐containing buffer) at a 1:4 (v:v) ratio to cross‐linked PF4 polypeptide reaction in MES buffer.

### PF4‐Polyanion HIT ELISA

Lifecodes PF4 IgG (Immucor) ELISA, an FDA‐approved in vitro diagnostic assay that employs PF4‐polyvinyl sulfonate (PVS) targets, was used according to the manufacturer's instructions. In brief, patient serum was incubated with PF4‐PVS coated platelets, stringently washed, and incubated with an alkaline phosphatase‐labeled anti‐human IgG antibody. After antibody incubation, p‐nitrophenyl phosphate (pNPP) substrate was added and colorimetric detection with optical densities of 405 and 492 nm was performed after 30 min.

### VITT ELISA

Cross‐linked PF4 was immobilized (0.5 µg well^−1^) on ELISA plates (Thermo Scientific) overnight at 4 °C. ELISA plates were washed three times with PBS pH 7.4 with 0.1% Tween‐20 and blocked with Superblock T20 (Thermo Scientific). Patient samples were added to the plate at 1:50 dilution for 1 h, and washed four times with PBS/0.1% TWEEN‐20. After a 45 min incubation with 50 µL of alkaline phosphatase‐conjugated goat anti‐human IgG Fc antibody (Jackson Immunoresearch) at a dilution of 1:5000, four additional washes were performed using PBS/0.1% TWEEN‐20. Assessment of colorimetric development was carried out using p‐nitrophenyl phosphate (pNPP) substrate, and the optical density (OD; 405 nm minus 492 nm) was recorded at 30 min. The positive cut‐off of the VITT ELISA was set at three standard deviations above the mean optical density of 50 healthy donor samples (0.341).

### Statistical Analysis

The datasets were obtained and analyzed using PSTrace 5 software to calculate the area under the SWV curves and determine the total charge. The normalized charge values (Q̅) were calculated using the formula (QS‐QNC)/(QPC‐QNC), where QS represents the charge of the clinical serum sample, QNC corresponds to the charge of the negative control (control serum), and QPC represents the charge of the positive control (HIT‐like antibodies or VITT‐like abs or VITT‐positive serum sample). Each data point represents the mean of two technical replicates, and in bar graphs, error bars indicate the standard deviation (SD). Statistical analysis and data visualization were performed using GraphPad Prism 6 (GraphPad Software, USA). Two‐tailed Mann–Whitney *U* tests were applied for all pairwise group comparisons. A significance level (α) of 0.05 was used (*p* < 0.05 considered significant). The sample size (n) for each group is provided in the corresponding sections.

## Conflict of Interest

A.P. reports pending/issued patents (Mayo Clinic, Retham Technologies, and Versiti Blood Center of Wisconsin), equity ownership in and serving as an officer of Retham Technologies, and equity ownership in Veralox Therapeutics.

## Author Contributions

D.F.C.A. and A.K. contributed equally to this work. The conceptualization, led by A.R., A.P., D.F.C.A., and A.K., established the foundation for the study. The methodology was developed and executed by D.F.C.A., A.K., S.L., A.M.G.S., K.G., E.M., and T.Q.N., with the investigation primarily conducted by D.F.C.A., A.K., and S.L., D.F.C.A. and A.K. were responsible for the visualization components. Funding acquisition and supervision for the project were handled by AR and AP. The writing process involved an original draft prepared by D.F.C.A., A.K., and A.R., which was subsequently reviewed and edited by D.F.C.A., A.K., S.L., A.M.G.S., K.G., T.Q.N., A.P., and A.R.

## Supporting information



Supporting Information

Supporting Information

Supporting Information

## Data Availability

The data that support the findings of this study are available from the corresponding author upon reasonable request.
